# The predictive validity of admission criteria for the results of clinical competency assessment with an emphasis on family medicine in the fifth year of medical education: an observational study

**DOI:** 10.1186/s12909-022-03293-y

**Published:** 2022-04-12

**Authors:** Thomas Kötter, Silvia Isabelle Rose, Katja Goetz, Jost Steinhäuser

**Affiliations:** 1 Institute of Family Medicine, University Medical Centre Schleswig-Holstein, Campus Lübeck, Ratzeburger Allee 160, 23562 Lübeck, Germany; 2 Institute of Social Medicine and Epidemiology, University Medical Centre Schleswig-Holstein, Campus Lübeck, Ratzeburger Allee 160, 23562 Lübeck, Germany

**Keywords:** Education, Medical students, Medical school admission, Criteria clinical competence, General practice

## Abstract

**Background:**

In many countries, the number of applicants to medical schools exceeds the number of available places. This offers the need, as well as the opportunity to medical schools to select those applicants most suitable for later work as a doctor. However, there is no generally accepted definition of a ‘good doctor’. Clinical competencies may serve as surrogates. The aim of this study was to compare medical students in Germany selected based either on their pre-university grade point average alone or based on the result of a university-specific selection procedure regarding their clinical competencies with an emphasis on family medicine in the later years of training.

**Methods:**

We used the ‘Allgemeinarztbarometer Ausbildung’ (Undergraduate Family Medicine Barometer), an instrument developed to assess clinical competencies with an emphasis on family medicine, to compare students in the pre-university grade point average admission-quota and the university-specific selection procedure admission-quota in the fifth year of training. Students were judged by their supervising general practitioners after a two-week practical course. Competencies were rated on a five-point Likert-scale (1 = ‘totally agree’ i.e. the student is very competent to 5 = ‘totally disagree’ i.e. the student is not competent at all).

**Results:**

We included 94 students (66% female). Students in the university-specific selection procedure quota (*n* = 80) showed better mean scores in every item of the Undergraduate Family Medicine Barometer. We found a statistically significant difference between the two groups for the item assessing communication skills (*M* [university-specific selection procedure quota] = 1.81, *SD* = 0.84 vs. *M* [pu-GPA quota] = 2.38, *SD* = 0.96; *t*[91] = -2.23, *p* = .03; medium effect size). Logistic regression revealed no statistically significant age or gender contribution.

**Conclusions:**

Despite the small sample-size, our results indicate, that students selected via an university-specific selection procedure show better communicative competencies in the later years of training.

**Supplementary Information:**

The online version contains supplementary material available at 10.1186/s12909-022-03293-y.

## Background

In many countries, the number of applicants to medical schools regularly exceeds the number of available places. The continuing high popularity of medical education offers the opportunity and entails the obligation for medical schools to select those applicants most suitable for the later work as a doctor.

In Germany, there are five applicants per study place at medical schools [1]. Recently, the statutory framework for medical school admission in Germany has been reworked following a judgement by the German Federal Constitutional Court [2]. The restructured legal requirements for the selection process include a quota of 30% selected solely based on the pre-university grade point average (pu-GPA; [3]). At least two pu-GPA-independent selection criteria have to be considered in addition to the pu-GPA in the university-specific selection procedures (60% of the study places). Ten percent of the study places are assigned based solely on pu-GPA-independent criteria. The question, however, whether university-specific selection procedures lead to ‘better doctors’ compared to pu-GPA-based selection, remains, to date, unanswered. Answering this question is hampered by the fact that, until today, there is no generally accepted definition of a ‘good doctor’.

Cognitive characteristics like subject knowledge, intellectual ability, and study habits are assessed by the pu-GPA. Consistently, pu-GPA show a predictive validity for medical school performance [4]. But are these characteristics aspects of a ‘good doctor’?

According to Parsons’ sociological analysis of the doctor-patient-relationship, the role of the physician is characterised by an unrestricted readiness to help (universalism), professional (medical) competence, functional specificity, affective neutrality and collectivity orientation [5]. Merton observed, that medical students acquire these characteristics by comparing themselves to and emulating ‘role models’ during their education [6].

Among others, these aspects of the physician role have been translated into a physician competency framework called ‘CanMEDS’ originally in 1996 and since then developed further for the use in competency-oriented medical education and specialty training programs [7]. The 7, partially overlapping, CanMEDS roles (Medical Expert, Communicator, Collaborator, Leader, Health Advocate, Scholar, and Professional) can be used to develop specific educational goals, as well as assessment tools for the evaluation of their achievement. Different country-specific frameworks around the world are based on the CanMEDS roles. They can be regarded as an important theoretical superstructure for the development of medical curricula that aim to pave the way for individuals to develop into ‘good doctors’. Consistently, a currently progressing restructuring of the medical curriculum in Germany has the competence orientation as one of the highest premises [8]. Thus, selecting those applicants with the highest potential to develop the desirable competencies for the medical profession seems to be more important than ever.

Different stakeholders, e.g. doctors, patients, nurses and students, have, however, different concepts of a ‘good doctor’ and rate or rank aspects connected to the ‘good doctor’ differently [9]. General interpersonal qualities, such as friendliness, politeness and empathy, as well as communication skills, such as attentive listening and understandable explanations are highly demanded among all stakeholder groups, especially patients. Medical competence, including clinical decision-making and a holistic view of medical problems, as well as ethics, are most important for doctors themselves. The latter category contains humbleness, honesty, confidentiality as well as the recognition of one’s own limitations. The first category is also rated highest by students, along with general interpersonal qualities and communication skills [10]. From the patients’ point of view, communication skills (including patient involvement) belong to the most important features of a ‘good doctor’ [9].

The ‘good doctor’ seems therefore to be made up of several different competencies, whose ideal proportions differ depending on the position in health care. Certainly, there are more aspects connected to the ‘good doctor’ than the cognitive characteristics assessed by the pu-GPA.

The question as to whether the development of competencies can be predicted more accurately by adding the assessment of non-cognitive characteristics to pu-GPA results during the selection for medical school has not yet been answered comprehensively. Considering the immense effort for university-specific selection procedures and the necessity to select those who are most likely to become ‘good doctors’, the question arises as to whether students selected by this kind of procedure are more likely to become ‘good doctors’, compared to students selected solely based on their pu-GPA. There are a few studies showing that selection procedures also based on desirable competencies rather than solely based on pu-GPA may be efficient in selecting applicants more likely to develop in a favourable way [11–15]. However, most of these studies compare students in different admission quotas only until the third year of undergraduate training. Schreurs et al. [15] showed students admitted via a university-specific, outcome-based selection procedure to outperform their initially rejected but lottery-admitted counterparts at the end of their clinical training regarding different CanMEDS roles. Sladek et al. [14] retrospectively matched different selection score results to competence assessments of interns in an Australian health network. They showed statistically significant associations between both pu-GPA and panel interview scores and workplace outcomes. In their systematic review, Ferguson, James and Madeley [16] found that selection interviews probably add useful additional information that has predictive power for relevant outcomes.

To our knowledge, however, studies are still scarce regarding the additional value of assessing non-cognitive characteristics during selection for medical school, especially for the German legislative context, that compare students in the later years of training.

Therefore, our objective was to compare students selected based either on their pu-GPA alone or based on the result of a university-specific selection procedure (outlined in brief below) regarding their clinical competencies in the later years of training.

## Methods

### Study design/setting

We conducted a cross-sectional observational study at Lübeck Medical School, a section of the public life-sciences oriented University of Lübeck, Germany. About 1,600 students are enrolled in the medical study program; 67% of them are female [17].

### Participants

We invited medical students in their fifth year of study during a seminar at the beginning of the semester for three consecutive semesters (October 2016, April 2017 and October 2017; *n* = 321 students). All students that gave written informed consent after a short presentation outlining the study were included. There were no exclusion criteria at this stage. However, we excluded individuals admitted at another medical school (or those who changed to Lübeck Medical School during the course of their studies) and via an admission quota other than based on pu-GPA or the university-specific selection procedure from the analyses.

### Outcome

Medical students who had given informed consent to participate were judged regarding their clinical competence by their supervising general practitioners after an obligatory two-week practical course in general practice in their fifth year of medical school. The fifth year of medical school in Germany is the last year of mainly theoretical medical education before the final ‘practical year’. During the time-period of the study, the staff of about 70 teaching practices supervised an average of three students per year.

We used the ‘Allgemeinarztbarometer Ausbildung’ (Undergraduate Family Medicine Barometer [UFMB]), an instrument developed to assess clinical competencies with an emphasis on family medicine based on the CanMEDS roles (see Table 1; [18]). The supervising general practitioners were instructed to rate the students at the end of their practical course and to give them feedback on the basis of their rating afterwards. The ratings were not converted into grades. Competencies were rated on a five-point Likert-scale (1 = ‘totally agree’ i.e. the student is very competent to 5 = ‘totally disagree’ i.e. the student is not competent at all).

**Table 1:** UFMB items

UFMB Item	I was able to convince myself that the student…
1	…has a good understanding of specific decision-making processes in family medicine
2	…can deal with diagnostic uncertainty
3	…can pick up the patient where he/she stands with his/her communication skills
4	…has a ‘holistic view’ of patients
5	…can involve patients in medical decisions in a participatory manner
6	…has acquired an attitude that allows ‘lifelong learning’
7	…is physically resilient for family medicine
8	…developed strategies against ‘burnout’
9	…is decisive in his/her work

The UFMB was part of a longer questionnaire containing 11 further items originally developed by Knorr et al. [19]. Results for other aspects of the questionnaire have been published elsewhere [20].

The general practitioners supervising students on their obligatory two-week practical course in general practice were informed about the study and made familiar with the UFMB, which they first used in the context of this study, in a 90-min CME-certified session in advance.

### Predictor

Outcome scores were compared between students admitted (at least 5 years earlier) to Lübeck Medical School solely based on pu-GPA (‘pu-GPA quota’) versus those selected based on the results of the university-specific selection procedure (‘university-specific selection procedure quota’). Other, much smaller selection quotas were the ‘waiting time quota’, the ‘German armed forces quota’ and the ‘foreign country quota’. Students in the university-specific selection procedure quota did apply directly for Lübeck Medical School after not meeting the pu-GPA or waiting time criteria. 240 direct applicants were invited for a 30-min face-to-face panel interview led by two faculty members and one student. The panel members were trained during general instruction sessions and an interview-training session. The interviewee was rated using a scoring sheet that also structured the interview with the five dimensions, i.e. motivation, knowledge about the course of study, social engagement, (self-)reflection and communication (max. score 30). The pu-GPA was converted into a score (max. 31) and added to the interview score in order to calculate a total university-specific selection procedure result [21]. Of the 240 interviewees, 120 were admitted to Lübeck Medical School. The interview scoring sheet did not change during the years we observed in our study. For further details regarding the university-specific selection procedure at Lübeck Medical School at that time, see Mommert et al. [22].

### Preventing selection bias

In order to reduce bias due to non-response, we offered all participants in the study and their supervising general practitioners a reward in terms of a book or food voucher for the amount of five Euros.

### Data management

Outcome data were matched to the admission quota by a data custodian using a Microsoft Excel 2010 table (Microsoft Corp., Redmond, WA, USA) and subsequently anonymised. Data sets were then imported into IBM SPSS Statistics for Windows Version 25.0 (IBM Inc., Armonk, New York, United States).

We excluded data from students that were admitted to LMS via any other admission quota (e.g., waiting time quota) and that were admitted to another medical school in the first place.

### Statistical methods

Data analyses were conducted using IBM SPSS Statistics for Windows Version 25.0.

We used t-tests to compare means of continuous variables and report results as means (M) ± standard deviation (SD). For gender, data were analysed using a chi-squared test and the results are reported as a percentage. In order to express bivariate correlations, we used Spearman’s ρ. We used binary logistic regression in order to control for age and gender and to further assess bivariate correlations, including variables with a *p*-value of > 0.25 in the bivariate analysis [23]. Due to the exploratory character of this study, we waived an adjustment for multiple testing and did not conduct any sensitivity analyses. All statistical tests were performed two-tailed with an α of 0.05. Effect sizes are reported using Cohen’s d. According to Cohen [24], we considered values of 0.2 small, 0.5 medium and 0.8 large effect sizes.

The study size was predefined by the number of students per class at Lübeck Medical School. In order to achieve a good compromise between homogeneity and study size, we recruited three complete, consecutive semesters.

Since all items of the UFMB were analysed separately, we did not exclude data sets with incomplete data. Numbers of data sets are reported for each UFMB item in Table 3.

This report was written in consideration of the STrengthening the Reporting of OBservational studies in Epidemiology (STROBE) statement [25]. See Supplementary file 1 for the completed STROBE checklist.

## Results

### Participants

We got informed consent of 165 students (51%) and UFMB ratings from 162 students. After exclusion of students admitted via another quota or at another medical school, we could include data of 94 students (66% female). Eighty students (85%) were in the university-specific selection procedure quota and 14 (15%) in the pu-GPA quota. For sociodemographic characteristics of the study participants, see Table 2.

**Table 2:** Sociodemographic characteristics of included students

	**all included students (*n* = 94)**	**university-specific selection procedure quota (*n* = 80)**	**pu-GPA quota (*n* = 14)**
*M* age (*SD*)	24.30 (1.53)	24.40 (1.56)	23.71 (1.27)
*n* female (%)	62 (66%)	51 (64%)	11 (79%)
*n* male (%)	32 (34%)	29 (36%)	3 (21%)

There were no statistically significant age and gender differences between the two quotas.

### Outcomes

Students in the university-specific selection procedure quota showed better mean scores in every item of the UFMB (see Table 3). Additionally, we found a statistically significant difference between the two groups for the item assessing communication skills (item 3; *M* [university-specific selection procedure quota] = 1.81, *SD* = 0.84 vs. *M* [pu-GPA quota] = 2.38, *SD* = 0.96; *t*[91] = -2.23, *p* = 0.03; d_Cohen_ = 0.67).

**Table 3:** Comparison of UFMB item scores between the admission quotas

UFMB item	*M* (*SD*) university-specific selection procedure quota	*M* (*SD*) pu-GPA quota	*t* (df)	*p*	d_Cohen_
1	1.75 (0.76)	1.93 (0.83)	-0.805 (92)	.42	n/a
2	1.83 (0.80)	1.86 (0.66)	-0.105 (90)	.92	n/a
3	1.81 (0.84)	2.38 (0.96)	-2.23 (91)	.03	0.67
4	1.99 (0.93)	2.25 (0.87)	-0.917 (88)	.36	n/a
5	2.11 (0.87)	2.15 (0.69)	-0.168 (83)	.87	n/a
6	1.65 (0.88)	1.83 (0.58)	-0.699 (84)	.49	n/a
7	1.35 (0.67)	1.71 (0.61)	-1.915 (87)	.06	n/a
8	1.96 (0.84)	2.00 (1.00)	-0.117 (54)	.91	n/a
9	1.99 (0.92)	2.07 (0.83)	-0.319 (92)	.75	n/a

### Predictors

We saw no statistically significant gender differences regarding the UFMB items except for item 7 (*I was able to convince myself that the student is physically resilient for general practice. M* [male] = 1.20, *SD* = 0.41 vs. *M* [female] = 1.51, *SD* = 0.75; *t*[86.59] = 2.51, *p* = 0.01; d_Cohen_ = 0.47). We saw no statistically significant correlations between age and UFMB item scores.

Logistic regression revealed no statistically significant age or gender contribution. The association between UFMB item 7 and the admission quota failed to reach statistical significance in the logistic regression analysis. However, we found a statistically significant association between UFMB item 3 (*I was able to convince myself that the student can pick up the patient where he/she stands with his/her communication skills*) and the admission quota (see Table 4).

**Table 4:** Logistic regression analysis for admission quota. Nagelkerke’s *R*^*2*^ = .20

Predictor	Range	Odds ratio	95% CI
Age	22–29	0.39	0.09–2.19
Gender	0 female	0.77	0.47–1.21
	1 male		
UFMB item 3	1–6	2.51	1.02–6.18
UFMB item 7	1–6	1.35	0.47–3.82

## Discussion

### Key results

In our cross-sectional study, we compared clinical competencies assessed using the Undergraduate Family Medicine Barometer during the fifth year of medical studies between medical students from different admission quotas (pu-GPA versus university-specific selection procedure). Students selected for medical education not solely based on pu-GPA scored significantly better regarding communication skills.

Communication skills are among the highest ranked or rated competencies of the ‘good doctor’ across all stakeholder groups [9]. Among the key concepts of the ‘Communicator’ role in the CanMEDS framework are attention to the psychological aspects of illness, empathy, a patient-centred approach to communication and shared decision making. Doctor-patient-communication can be seen as a means to translate Parsons’ above mentioned characteristics of the physicans’ role [5] into good patient care. Effective communication can improve not only patient satisfaction, but also their adherence, safety and medical outcomes [26]. Especially in the family medicine setting, physician communication is not limited to the interaction between physicians and patients, but also includes communication with patients’ families, caregivers, and colleagues. Physicians’ self-efficacy regarding communication skills seems to be associated with a lower risk of burnout [27].

Thus, the assessment of non-cognitive characteristics, amongst them communication skills [22], in the context of the university-specific selection procedure at Lübeck Medical School seems to be effective regarding the goal to select students with a high potential to develop into ‘good doctors’.

Our results are in line with the results of earlier research on the predictive validity of selection procedures employing not only pu-GPA, but also non-cognitive characteristics. In addition to the studies discussed above [11–16], the multiple mini-interview (MMI)-scores of medical students were found to predict the communication skills assessed in an objective structured clinical examination after one and a half years of medical education [28]. MMIs are claimed to be a valid, cost-effective alternative to conventional admission interviews and suitable for the assessment of interpersonal skills (i.e., empathy and communication skills). Evidence for their predictive validity regarding interpersonal skills from prospective, longitudinal studies is still scarce [29], as for traditional selection interviews [14, 15].

Therefore, our results add important information to the discussion about how to select applicants for medical education in a way that it improves its ‘Outcome’, i.e. leading to ‘good doctors’ with desired skills.

### Strengths and limitations

This study has several strengths and limitations. To our knowledge, this is one of the first studies linking the admission quota for medical education to an interpersonal skill (i.e. communication skills) assessed in the later years of medical training, just before the final undergraduate year. Since other studies conducted in Germany mainly focus on admission processes employing MMIs, linking the results of an admission procedure based on traditional selection interviews is, to date, unique. We chose the fifth year of undergraduate training for the assessment, because at Lübeck Medical School, students attend a two-week internship in a general practice in this year. The rating by general practitioners, who come to know the students for these two weeks during their consultation hours/home visits and teach them in a one-on-one setting, should therefore be comparatively valid, as no other supervisor spends this amount of time with individual students.

Due to the relatively small sample size, our results have yet to be interpreted with caution. The size of our study limited the possibility of more in-depth analyses, e.g. to control for multiple potential confounders. However, we did control for age and gender as perhaps the most important potential confounders for the outcome communication skills. Our study is single-centred. This clearly limits the generalisability of our results, since the nature of the university-specific selection procedure at Lübeck Medical School is unique. However, the age and gender distribution matches the distribution in the whole student population (67% female; [17]) and resembles nationwide distributions [30, 31].

Another limitation of our study is the use of a, to date, not broadly used instrument (the UFMB) for the assessment of our outcomes in the context of medical education. However, the instrument is already established in the context of specialist training [32, 33] and we validated it for use in the present context [18].

### Implications for research and practice

Our finding, that medical students selected not solely based on their pu-GPA score higher with regard to communication skills in their fifth year of study has to be confirmed in larger studies. These studies should employ other instruments for the assessment of this skill, e.g., objective structured clinical examination stations designed to assess interpersonal skills as a suitable context for the objective assessment of communication skills during medical education.

Our study could be a signal and motivation for other universities to employ similar studies for the evaluation of their often resource-intensive university-specific selection procedures. Medical schools with a higher number of study places might have better opportunity to conduct in-depth analyses due to larger sample sizes. Especially after the introduction of the so-called ‘Landarztquote’, an admission quota reserved for students that commit themselves to working as a general practitioner in a rural area after medical school, in several federal states in Germany, an established tool for the evaluation of this new admission quota could be of good use.

In light of the above cited studies and our own results, we encourage medical schools to use any legislative leeway for a more global assessment of their applicants rather than to focus on the pu-GPA.

In order to enhance communication skills, assessments could be employed at the beginning/in the first years of medical studies and repeated later on, accompanied by tailored interventions to improve these skills [34].

## Conclusion

Despite the small sample size, our results indicate, that students selected based on the results of an university-specific selection procedure show better (communication-related) competencies in the later years of training (medium effect size). Thus, it may be preferable to select medical students not only based on their pu-GPA, but also using additional selection criteria. To our knowledge, this is the first study showing differences between students selected for medical school by different criteria in the later years of medical education. Larger scale studies are necessary to confirm these results.

## Supplementary Information

**Additional file 1. **

## Data Availability

The datasets used and/or analyzed during the current study are available from the corresponding author on reasonable request.

## Abbreviations

M: Mean
MMI: Multiple mini-interview
pu-GPA: Pre-university grade point average
SD: Standard deviation
STROBE: STrengthening the Reporting of OBservational studies in Epidemiology
UFMB: Undergraduate Family Medicine Barometer
