# Supplementary material for: The predictive validity of admission criteria for the results of clinical competency assessment with an emphasis on family medicine in the fifth year of medical education: an observational study
Source: BMC Med Educ. 2022 Apr 12;22:269. doi: 10.1186/s12909-022-03293-y (PMC9003966; doi:10.1186/s12909-022-03293-y)
Supplement: Supplementary file 1 — Additional file 1. [file 12909_2022_3293_MOESM1_ESM.docx]

**Supplementary file 1: STROBE checklist**

| **Item No.** | **Recommendation** | **Manuscript section** |
| --- | --- | --- |
| **Title and abstract** | | |
| 1a | Indicate the study’s design with a commonly used term in the title or the abstract | Title page |
| 1b | Provide in the abstract an informative and balanced summary of what was done and what was found | Abstract |
| **Introduction** | | |
| 2 | Explain the scientific background and rationale for the investigation being reported | Background |
| 3 | State specific objectives, including any prespecified hypotheses | Background |
| **Methods** | | |
| 4 | Present key elements of study design early in the paper | Study design/setting |
| 5 | Describe the setting, locations, and relevant dates, including periods of recruitment, exposure, follow-up, and data collection | Study design/setting,  Participants |
| 6 | Give the eligibility criteria, and the sources and methods of selection of participants | Participants |
| 7 | Clearly define all outcomes, exposures, predictors, potential confounders, and effect modifiers. Give diagnostic criteria, if applicable | Outcome, Predictor |
| 8 | For each variable of interest, give sources of data and details of methods of assessment (measurement). Describe comparability of assessment methods if there is more than one group | Outcome, Predictor |
| 9 | Describe any efforts to address potential sources of bias | Preventing selection bias |
| 10 | Explain how the study size was arrived at | Statistical methods |
| 11 | Explain how quantitative variables were handled in the analyses. If applicable, describe which groupings were chosen and why | Data management |
| 12a | Describe all statistical methods, including those used to control for confounding | Statistical methods |
| 12b | Describe any methods used to examine subgroups and interactions | Statistical methods |
| 12c | Explain how missing data were addressed | Statistical methods |
| 12d | If applicable, describe analytical methods taking account of sampling strategy | n/a |
| 12e | Describe any sensitivity analyses | Statistical methods |
| **Results** | | |
| 13a | Report numbers of individuals at each stage of study, e.g. numbers potentially eligible, examined for eligibility, confirmed eligible, included in the study, completing follow-up, and analysed | Participants |
| 13b | Give reasons for non-participation at each stage | Participants |
| 13c | Consider use of a flow diagram | n/a |
| 14a | Give characteristics of study participants (e.g. demographic, clinical, social) and information on exposures and potential confounders | Participants |
| 14b | Indicate number of participants with missing data for each variable of interest | Outcomes |
| 15 | Report numbers of outcome events or summary measures | Outcomes |
| 16 | Give unadjusted estimates and, if applicable, confounder-adjusted estimates and their precision (e.g., 95% confidence interval). Make clear which confounders were adjusted for and why they were included | Outcomes, Predictors |
| 17 | Report other analyses done, e.g. analyses of subgroups and interactions, and sensitivity analyses | n/a |
| **Discussion** | | |
| 18 | Summarise key results with reference to study objectives | Key results |
| 19 | Discuss limitations of the study, taking into account sources of potential bias or imprecision. Discuss both direction and magnitude of any potential bias | Strengths and limitations |
| 20 | Give a cautious overall interpretation of results considering objectives, limitations, multiplicity of analyses, results from similar studies, and other relevant evidence | Conclusion |
| 21 | Discuss the generalisability (external validity) of the study results | Strengths and limitations |
| **Other information** | | |
| 22 | Give the source of funding and the role of the funders for the present study and, if applicable, for the original study on which the present article is based | Funding |
